# A Practical Treatise on the Diseases of Children

**Published:** 1843-07-01

**Authors:** 


I. A Practical Treatise on the Diseases of Children. By *James Stewart*, M.D. New York, 1841. 8vo. pp. 544.

II. The Diseases of Children. By *G. A. Rees*, M.R.C.S. London, 1841. 12mo. pp. 300.

III. The Maternal Management of Children in Health and Disease. By *Thomas Bull*, M.D. London 1840. 12mo. pp. 302.

IV. Conseils aux Meres sur la Maniere d'élever les Enfans, &c. Par *Al. Donné*, M.D. Paris, 1842. 12mo. pp. 295.

It is a common, although we think a very erroneous, opinion, that the treatment of the diseases of infants and children is, on the whole, more difficult and unsatisfactory than that of the diseases of adults. Certain it is that the former branch of medical practice is much less generally well understood than the latter; and that many a physician, of admitted ability and skill in the management of the one, is yet egregiously deficient in a due knowledge of the other set of maladies. It is objected that, as young creatures cannot give us any distinct information with respect to the seat and nature of their sufferings, we are necessarily left in considerable doubt, and must therefore be often puzzled what opinion we are to form, and what treatment we should adopt to relieve them. The objection is to a certain extent quite true; but then, to counterbalance this source of ambiguity, let us remember that the character of children's diseases is very generally much more simple and uncomplicated than that of the diseases of adults; moreover, that we have not to deal with the disturbing and perplexing influence of the mind or its emotions of hope and fear and feverish anxiety; and lastly, that the conservative and restoring energies of the constitution are far greater in early life than in later years. Not only is the nosological catalogue of children's diseases comparatively very limited in point of number, but there is also a much greater uniformity in the character and course of the same disease, as it occurs in different individuals and at different seasons; and hence it comes to pass that we have not occasion for any great variety of remedies in the treatment, and that the pharmacopœia of infantile medicine, so to speak, is on the whole very small. With the possession of calomel and the hydrargyrum cum cretâ, of ipecacuan and Gregory's powder (magnesia, rhubarb and ginger), of castor oil, salts, and nitre, not forgetting a due supply of leeches, the experienced practitioner will feel himself abundantly provided in ninety, nay perhaps in ninety and nine, out of every hundred cases that come under his notice. Certain classes of medicines are but seldom called into requisition at all. For example, how rarely have we any occasion for the use of Tonics in children's complaints: all that the young creatures, when much weakened, usually require for the recovery of their strength is a supply of light nourishing food after the stomach and bowels have been duly prepared for its reception. Then again, with respect to the classes of Narcotics, Antispasmodics, Diuretics, Alteratives, &c. it is certainly not often that we need resort but to the very simplest kinds of these medicines; such as a little syrup of poppies, sal volatile, and sweet nitre. A vast deal of harm is almost daily done by the use of stimulants and sedatives in children's complaints; and it is well known that the soothing and cordial syrups of the nursery, *Godfrey's*, *Daffy's*, *Dalby's*, &c. elixirs, have given a regular *quietus* to not a few of the young sufferers.

To judge aright of children's diseases, the medical man should attentively consider what are the peculiarities of their constitution compared with those of the adult system. This is a subject that has been very seldom dwelt upon in the very numerous works on this department of medicine with the attention that it deserves; and yet a right knowledge of this point seems to us to lie at the very foundation of any rational or successful system of infantile therapeutics. Let us for a moment consider the question; and first of all let us ask ourselves, what are the main peculiarities in the organisation of the child? They appear to us to be two fold: viz. on the one hand, a preponderating size of the head and consequently an extra amount of blood sent to it; and, on the other, an extreme irritability and sensitiveness of the dermoid and mucous surfaces. The truth of these simple propositions is too obvious to require any illustration; and therefore, as our space is necessarily limited, we shall say nothing on this head, but proceed at once to enquire what are the practical conclusions to be drawn from their consideration.

The *first* one naturally leads to this most important therapeutic rule, that the head of the child should be uniformly kept exceedingly cool, in order to prevent any congestion or over-action in the vessels of the cerebrum.

From the very large quantity of blood sent to this organ, there must needs be a greater than usual tendency in early life to irritation and plethora within the head; more especially during the whole of that period when the early teeth are in a state of development and growth. Does not this consideration therefore at once suggest the utility of keeping the child's head exposed, and uncovered with warm caps, heavy bonnets, &c. and, in many cases, of wetting the scalp frequently with cold water, or any simple refrigerant lotion, while the rest of the body and more especially the feet are kept warm, with the view of diverting the course of the blood in another direction? Such a simple expedient as this, coupled with the use of an appropriate diet, of cooling laxatives, and of a leech or two occasionally to the temples if necessary, will often suffice to check the development of much future cerebral mischief, and enable the little sufferer to pass through the dangerous period of dentition with safety and comfort. And then what is the hygienic inference that we draw from the *second* position that we laid down; viz. the great tenderness and irritability of the skin and of the mucous passages? Is it not that the former should be duly protected against the inclemencies of a changeful climate, and that the latter—we allude more particularly to the bowels—should not be irritated with stimulating or indigestible ingesta? Hence we are led to perceive the necessary importance of sufficient clothing, and of constant attention to the proper diet of children; and yet we see these simple directions transgressed over and over again among all the classes of the community, and often, too, under the very eyes of medical men.

As we wish to call the attention of our readers to the very useful works of Dr. *Bull* and M. *Donné*, we shall take the present opportunity of making some remarks on the *hygiene* of infant life, blending the results of our own experience with the observations of these gentlemen.

It is a shrewd remark of M. *Donné* that the management of an infant's health should commence, if we may so speak, from before its birth. The future vigour of the offspring depends so much on the health of the parent during the period of her pregnancy, that medical men cannot too often or too seriously remind their patients of the great importance of attention to this circumstance.

Most mothers seem to be not at all aware of its truth; else they would surely act on many occasions very differently from what we observe them to do.

Without entering minutely into the question of the management of women during the period of their gestation, we may here allude to one particular only—but this is a very important one—which is far too much overlooked in the present day; and yet to the neglect of which may often be traced the feebleness and bad health of the child after birth—we mean the importance of their taking regular exercise in the open air every day, as long as circumstances will possibly allow. The injury that is often imperceptibly—on this very account, the more dangerous—done to the mother's constitution by remaining, as is too frequently done, whole days and even weeks, if not months, within doors—perhaps in close confined rooms too—is immense. If spoken to on the subject, your patient will probably tell you that she feels very well, and that she is not sensible of any inconvenience to her health by the confinement. This may be all very true; she may not experience any ill effects at the time; but let her rest assured that nature's laws and nature's precepts cannot be disregarded without some future detriment ensuing either to herself or to her offspring. Does any plant or animal, we may ask, ever thrive as well under constant cover, as when exposed to the free air of heaven? certainly not; then why suppose that there can be any exception in the case of human beings. There is a simple, but very sound rule for our guidance on most points connected with the preservation of health, which deserves more attention than is usually given to it, and it is this: "Whatever does no good, does harm." True maxim! applicable to the discipline alike of mind and body.

By much in-door confinement, as M. *Donné* very truly remarks, the mother's system is weakened and rendered irritable, a feverish restlessness is induced, her appetite decays or becomes irregular and capricious, and her general comfort is often seriously marred during the remainder of her pregnancy. She thus becomes far less favourably prepared to pass through the pains and anxieties of child-birth without injury; she is rendered more liable to many puerperal complaints; and, if there was no other inconvenience, she is much less fitted, than she might otherwise have been, to be a good nurse to her child. The health of the latter so intimately depends first upon its own actual condition when born, and secondly on the quality of the food provided for it, that it must be quite unnecessary to enter upon any argument to prove that, unless the mother be thoroughly healthy and prepared, as Nature designed, to supply a due supply of sound wholesome food, we cannot reasonably expect that her offspring can be vigorous and robust. As well might we look for pure fair water to spring from a brackish well, or a thriving shrub to shoot out from a dry sandy soil. The great secret therefore of rearing a child well is unquestionably, in the first place, to see that the mother's health is kept in a sound and wholesome condition, so that there may be a regular and sufficiently, but not an over, abundant supply of wholesome milk. Now Nature has been exceedingly benificent in her arrangements for this most important end, and it is only because we choose to deviate from them, and to act, so to speak, independently and often too at open variance with her wise appointments, that there are so many bad and incompetent nurses in the world. Is it not a matter of daily observation that a woman's health is seldom so uniformly good as during the whole period of her suckling? and do we not continually meet with cases where the usual course of complaints and diseases is obviously arrested by an All-merciful Providence for many months, in order that the helpless infant may be supplied with its natural food for a sufficient time to enable it to weather the subsequent trials of its orphaned life? We mention these circumstances to shew that Nature has done almost everything ready to our hands, and that, if we only did not wilfully thwart her most obvious intentions, we should seldom hear of the great difficulties, and often dangers too, that both mother and child are subject to during the period of suckling.

Let us briefly consider some of these difficulties, and endeavour to find out whence they arise and how they may be most advantageously treated.

The first that we allude to is the very common and ignorant practice of encouraging an excessive secretion of the milk, and the consequent suckling of the infant far more frequently than is necessary or wholesome. It is a very common opinion that a woman, while she is nursing, requires not only a very full diet of nourishing food, but also a good large allowance of some stimulating, usually fermented, liquor. "The only result," says Dr. *Bull* very truly, "of this plan is, to cause an unnatural degree of fulness in the system, which places the nurse on the brink of disease, and which of itself frequently puts a stop to the secretion of the milk, instead of increasing it. The right plan of proceeding is plain enough; only let attention be paid to the ordinary laws of health, and the mother, if she have a sound constitution, will make a better nurse than by any foolish deviation founded on ignorance and caprice."

Most suckling women in this country would consider themselves half-starved, if they were not allowed one or two pints of ale or porter per diem; and yet, might we not ask them how do their sisters thrive in other lands, where these Castalian beverages are unknown? Of this they may be assured that, if a sufficient quantity of light wholesome nourishment be taken at regular intervals, and due attention be paid to other hygienic rules, and especially to that of taking daily exercise in the open air, there will be no deficiency of milk, and the milk moreover will be of a better kind at the same time. How many a woman among the poorer classes in this country ruins her health by the common practice of forcing the secretion by drinking large quantities of beer of some sort or another! Now what is the usual effect of this practice? The appetite for solid food diminishes, the strength fails, the woman feels a sinking at the pit of the stomach, and a sense of dragging pain in the loins, and all the while the poor creature, attributing her every suffering to mere weakness, imagines that, if she only drinks more beer, she will be better. True it is, that her sufferings arise from weakness; but unfortunately she is pursuing the very plan to increase it. If she would abandon, for a time at least, the malt liquor, and live upon good wholesome meat and broth, and not over suckle her infant, we should see fewer instances of broken constitutions among the mothers, and of shrivelled decrepid and half-fed children at the breast.

Even among women in the higher and better educated classes of society, there is a vast deal of prevailing error, as to how they should manage their own diet, and regulate the suckling of their infants.

Many a young mother, on finding that for the first few weeks after confinement she has a great flow of milk, naturally supposes that she must be an excellent nurse, and that, if her baby does not thrive, it cannot possibly be from any fault on her part. The fault—if *fault* it can be called—arises from an affectionate over-desire to do all that she thinks likely to benefit her child. She therefore encourages what is popularly called a frequent 'draft in the breast;' and nothing certainly does this so effectually as consuming large quantities of malt liquor. But it is right that she should know that the mere abundance of the milk for the first month or so is by no means a sure guarantee that it will continue so; or, if it does, that the milk will necessarily be wholesome and nutritious. Most women—even those who afterwards may be obliged to give up suckling from the deficiency of the secretion—have a tolerably abundant supply for the first few weeks. Every thing, be it observed, at this time, has a tendency to promote it;—the heated room, the warm clothing, drinking large quantities of fluid, want of exercise, &c. But then, as the woman begins to resume her former habits, and recurs to her ordinary diet and *regime*, the secretion of the milk often becomes much less abundant, while the wants of the child become greater; or, if the quantity remains sufficient, —probably in consequence of the nurse living chiefly on milk, liquor, and other fluids—the quality is perhaps poor, watery, and unnutritious. The practised eye will at once suspect this condition of the milk, partly from the looks of the mother, and in part from the general appearance and health of the child. If the former is pale and languid, if the white of the eyes be blueish, and the lips and tongue have lost their natural redness; if she complain of weakness in her back and limbs, and of a sense of faintness and sinking at the pit of the stomach—this last, we should say, is one of the most trustworthy of all the symptoms—especially after suckling, we may be pretty confident that the milk, however abundant, is watery and thin. A very common accompaniment of such symptoms in nurses is the existence, to a greater or less degree, of what is well known to them by the name of weakness; *i. e.* leucorrhœa.

When the milk is in this state, the child is observed not to thrive so well as might be expected, considering the frequency with which it takes the breast, and the abundant supply of the milk;* it is feverish, uncomfortable, and often crying; its bowels are very generally relaxed, and every now and then much purged; they are always more or less flatulent, and the infant is often teased with griping pains; the stools are seldom healthy; and the sleep is short and easily disturbed.

A much less frequent condition of the milk that we have to provide against is the reverse of all this—viz. the over-richness of a nurse's milk. This is very rarely the case with a mother suckling her own child; but every now and then we have occasion to observe its effects when young healthy nurses from the country are brought to town for the purpose of suckling the children of the higher classes. When the milk is too rich for an infant's stomach, it generally rejects more or less of it immediately after suckling; and, if it does not, it will be observed to be uncomfortable, and every now and then making an effort to retch for some time afterwards. As this condition however of the milk is certainly but of rare occurrence, we need say nothing more about it at present.

The practical inference from all that we have said is, that the diet of a nurse should not differ in any great respect from what she is ordinarily accustomed to, and should consist of fresh and easily-digested animal food

* Indeed the appetite or craving of the infant for the breast is usually greater than usual; and for this very simple reason, that the milk, from being less substantial and nutritious than it ought to be, remains but a short time in the stomach, and passes without much change into the bowels. Here therefore is a simple sign—viz. the more frequent than usual desire of the infant for the breast —which may lead the nurse to suspect that her milk is defective in nutriment.

once or twice a day, with a due allowance of bread and vegetables, and a moderate quantity of sound well-fermented malt liquor. As long as the appetite and digestion continue healthy, there is no objection to this beverage; but if, after a time, these begin to fail, then—instead of increasing the quantity of the ale or porter drunk, in order to compensate for the deficiency in the amount of solid food taken—the use of it should be at once suspended for a few days, until the functions of the stomach be regulated and its energy restored.

The mother, indeed, will probably ask us, how is she to manage her baby; for, without her beer, she is sure that she will have no milk at all. This question leads us to the consideration of another one, of no trifling importance in the *hygiene* of infant life, to wit:—

*Ought a child to have any other food but the breast milk for the first five or six months?*

Like every other question in medical practice, this one does not admit of an answer applicable to all instances, and proper under all circumstances. The following remarks however will, we trust, assist the reader to determine the right course to be pursued in each individual case.

When the nurse is uniformly healthy, and when the child evidently thrives on her milk, gaining gradually more and more plumpness and strength, we may feel assured that it does not require any other sort of food. But let it be remembered that this fortunate state of things is by no means of very frequent occurrence—more especially among the residents of large towns—and that every deviation from the state of perfect health on the part of the nurse, is necessarily attended with a certain amount of change in the secretion of the breasts.

Now there are a thousand little things which are apt to do this. For example, a slight indigestion, any derangement of the bowels, more than ordinary fatigue, a sudden fright, over-anxiety, the receipt of bad news, and even excessive joy—these and many other troubles to which suckling women are exposed, all tend to affect the qualities of the milk. It is astonishing how little an affair will be sometimes followed by an alteration in the condition of this secretion—manifested, indeed, not in the health of the mother, but in that of the infant. Every experienced medical man must have seen cases where griping and purging were induced in an infant, in the course of a few hours after the nurse had taken one glass of port wine; and where its very life has been brought into jeopardy from the anxiety of the mother—in consequence, perhaps, of the sickness of another of her children.

Among the lower classes, we feel quite assured that three-fourths of infantile diseases may be attributed to the unwholesome state of the milk, induced by the use of impure and *drugged* malt liquors.

There is one cause—not a very common one, indeed, but sufficiently so to deserve notice—that is almost invariably accompanied with a derangement of the milk, and consequently of the infant's health—we allude to the occurrence of the periodical monthly indisposition in the nurse during the time of suckling. The intimate sympathy between the womb and the breasts is shewn by a variety of circumstances, but by none more obviously than by this circumstance. It may be laid down as a very general truth, that whatever causes a diversion of the blood towards the former organ will induce a diminution in the secretion of the latter.

But not only is the quantity of the secretion diminished; it is almost invariably altered in quality. Hence, in nineteen out of twenty cases, where the nurse is apt to be *unwell* during suckling, the infant will suffer griping pains in the bowels, purging, sometimes fits, &c.—all which symptoms clearly shew that they are attributable to the food that is administered. We know, indeed, that some women will tell us that their baby does not seem to be at all affected by the cause now mentioned; but the medical man may rest assured that such is very rarely the case, and that in by far the majority of instances the very reverse holds true.

Let it not, however, be supposed that we should recommend a nurse to discontinue suckling altogether for this cause alone; but we do say that the less the child takes the breast under such circumstances the better, and that it should be fed chiefly by hand, as long as the menstrual discharge continues.

The inference we draw from these considerations is, that it will be prudent, even under the most favourable circumstances, not to trust to the breast *alone* in the rearing of a child; but to give occasionally such light food as may be a convenient substitute for the milk. Like the wise general, who will never commence a campaign, or even enter upon a single action, without providing a safe mode of retreat for his troops—knowing full well how some casualty, which no sagacity could foresee, may occur to baffle his most confident hopes—the physician will not be willing to trust all to the contingent health of the nurse, which is apt to suffer from such a variety of uncontrollable events. It is not because the infant, for the first four or six months of its life, has need of more nourishment than what a healthy nurse can supply, that we recommend the early use of other food at the same time;—but only that it may not suffer from the sudden change of diet which many circumstances may render necessary. And has not beneficent Nature herself suggested a useful lesson to us on this very point, in the working of those instincts that she has implanted in the lower animals to guide them in all their doings? How soon does the lamb begin to crop the herbage by the side of the ewe! and do we not see the pup and kitten trying to lap food, before they can well hold themselves erect upon their legs? We may be assured that we shall seldom err, if we but follow *the promptings of Nature's directions on this as well as on a variety of other topics connected with the preservation of health.*

*We proceed now to another question:—*

*How often should the Child be fed in twenty-fours?*

This is unquestionably one of the most important rules of all, in the management of infants, to be rightly determined; and we are glad to find that Drs. *Bull* and *Donné* entirely agree with each other in the precepts which they give under this head. The former says—

"At the expiration of a week or so it is essentially necessary, and with some children this may be done with safety from the first day of suckling, to nurse the infant at regular intervals of three or four hours, day and night. This allows sufficient time for each meal to be digested, and tends to keep the bowels of the child in order. Such regularity, moreover, will do much to obviate fretfulness, and that constant cry, which seems as if it could be allayed only by constantly putting the child to the breast." 8.

He afterwards adds, "in reference to night-nursing, I would suggest suckling the babe as late as ten o'clock p. m., and not putting it to the breast again until five o'clock the next morning. Many mothers have adopted this hint, with great advantage to their own health, and without the slightest detriment to that of the child. With the latter it soon becomes a habit; to induce it, however, it must be taught early."

M. *Donné* suggests that, during the first few weeks, the breast may be given every two hours or so, unless indeed the child sleeps a great deal, and then it requires to be applied less frequently: afterwards, once every three or four hours is amply sufficient. He very justly remarks that, as a matter of course, no exact or mathematical rules can be laid down on such a subject, and that a good deal must always be left to the discretion of the nurse. He expressly condemns the foolish practice of applying a child to the breast, whenever it begins to cry, and points out with great good sense the importance of having regular and stated hours for feeding.

As we expressed our own opinions on this subject in a late Number—vide Medico-Chirurgical Review for October, 1842, p. 566—we shall not do more at present than offer two or three remarks on some of the evils of overfeeding.

By far the greater number of infantile ailments arise from some disorder of the bowels, occasioning griping pains, vomiting, feverish restlessness, and not unfrequently 'inward fits' and convulsions. These symptoms are almost invariably owing either to the unwholesome quality, or to an excessive quantity, of the food administered. The evils of *stuffing* (to use a phrase more expressive than elegant) a child are soon sufficiently apparent. Often, as is well known, does Nature get rid of the excess by inducing vomiting of the superfluity; and mothers will sometimes pride themselves as being capital nurses when their infants give this most satisfactory sign of not being stinted of their allowance. Unfortunately the practice has much greater inconveniences than its unseemliness. The digestion of the food that is retained is almost always more or less interfered with, when the stomach has been once too full. It (the food) is not so readily exposed to the action of the gastric juice—the great agent in the process of digestion—nor does it favourably undergo those changes, which are necessary to fit it to become absorbed into the system and assimilated with the blood. This is not the only evil that is apt to follow; for the food, when imperfectly digested, will inevitably cause irritation of the bowels, as it passes along their highly sensitive surface, giving rise to griping pains, inward fits, and purging. Every nurse knows well that the alvine motions often exhibit portions of the curd of the milk quite unchanged. Is not this a most convincing proof that the child has swallowed more than it has been able to digest?* Diarrhœa in infants is the result as

* We may here mention one very simple, but most valuable, means of judging of the wholesomeness of food, especially in the case of children. Whenever any often of over-feeding, as of the unwholesome quality of the milk or other food that has been given. Nature acts the part of the kind physician; she sets up an action to get rid of what is offending the bowels; and often little more is required to check the complaint than merely to restrict the suckling to certain intervals of time.

*The Sleeping of Infants.*

Ought an infant to sleep with the nurse? is a question very intimately connected with the one of which we have just been treating. All medical men will, as a matter of course, disapprove of the too common practice of a child being put to the breast four, five, or six times during the course of a night; and yet this practice cannot well be avoided, if the little creature sleeps with its nurse. The mere smell, arising from the mamma constantly near it, provokes a most unnecessary frequency of returning appetite; and if there was no other reason against it, this alone would be a sufficient one to condemn the practice of the mother and child sleeping together. For the first month or six weeks, while the young infant has but comparatively a feeble power of maintaining its own heat—a fact that has been so beautifully proved by the researches of *Edwards*—this may be proper; but after that time, it should be accustomed to sleep in a little bed or cot by the nurse's side. It is of more consequence to women in humble life than to those in more easy circumstances to attend to this advice, as it is of especial importance to the former that their sleep should not be unnecessarily disturbed. No woman can be a good nurse, who has not from six to eight hours good sound sleep every night. She may indeed wake once, or twice at most, to give suck to her infant during this time; but even this may be unnecessary after the second or third month, as a child will often rest quietly itself without requiring food from eleven at night to five or six next morning—provided it has been accustomed to be fed only at stated intervals, and to sleep by itself. M. *Donné* has some very sensible remarks on the inconveniences of accustoming children to fall asleep in the arms or on the knees of their nurse; the earlier that they are reconciled to the use of the cradle, assuredly the better.

So much for Food and Sleep; and now a few words on

*Air and Exercise.*

There are certain months of the year in this climate in which it is wiser, as a general rule, to keep infants within doors than expose them to the inclemencies and vicissitudes of the weather. It is to be remembered, as we have already mentioned, that their bodies have a much feebler power of generating heat than those of grown-up persons; and the circumstance moreover of their being merely passive creatures, incapable of taking such

---

substance, or portion of it, passes through the bowels unchanged, we may be assured that it is indigestible, and should be prohibited. Hence cheese, the white of hard-boiled eggs, dried currants, &c. are certainly among the worst articles that can be given to a child. Many of the most dangerous cases of the *slow*, or remittent, fever of children are owing to an accumulation—which has been going on for a length of time—of such matters, along with the depraved secretion of the bile and the intestinal mucus in the bowels.

exercise as may stimulate the muscular and circulatory systems, renders them the more obnoxious to the injurious effects of undue exposure. If kept within doors, the nursery room should be large, lofty and cheerful; and, whenever the weather will permit, the windows may be thrown open. It is impossible to lay down any specific directions applicable to all cases, as much will depend upon a variety of circumstances touching the age of the child, its vigour and healthfulness, &c. &c.; but a well-instructed medical man can never experience any difficulty in advising what should be done. Unquestionably as a general rule, young children cannot be too much in the open air, whenever the weather is favourable; it is almost as necessary to them, as a means of vigorous health, as a due supply of wholesome food. Let us take a hint from what we observe in our daily walks.

Observe how all young creatures delight in the cool air of an open field, and how they pine when cribbed up in a cage or any close place within doors. It is just the same with our own infants; they cannot express, except by dumb signs, their satisfaction; but whoever is in the habit of watching their movements, from the earliest period of life, must have often observed the pleasure which they seem to feel in being exposed to the refreshing breath of a Summer's breeze. Their skin, it should be remembered, is greatly more sensitive to the action of the air, and indeed to all outward impressions, than that of an adult. From its being very much thinner, and also exceedingly vascular, the circulation on the surface of the body has been thought by many to undergo a change somewhat similar to that which takes place in the lungs; the blood thus becoming more highly oxygenated in the superficial vessels, and all the energies of the system being thereby greatly increased.

However this may be, it is quite certain that young children suffer much more from confinement in a close atmosphere than grown-up persons; and it can, therefore, require no further argument to prove the great importance of the precept we have been endeavouring to inculcate.

Independently of these direct advantages to an infant from being much out in the open air, there are others which, though not so immediate and obvious, are well worthy of notice. Often, very often, it will be found that, when all means have failed within doors of lulling a child asleep during the day, the nurse has only to walk up and down a garden or field with it for a few minutes, and the object is at once gained. Who has not experienced the influence of Nature's sounds in soothing the mind, and inducing slumber, when this 'best balm' had been chased from the pillow?

And let it not be imagined that the infant is not as much affected by them as we are in after-life. It is probably much more so; and for this very simple reason, that the mind is not yet developed; the young creature is a being merely of sensation and perception, easily influenced by all outward impressions, and especially by those which act upon the senses of touch and hearing. The cool breezy air, the rustling of the trees, the hum of the insect, the chirrup of the bird, are all, we may be assured, perceived by the young being long before it can give any utterance to its joy, even by inarticulate sounds; and the wise nurse will therefore often gladly avail herself of these sweet provocatives of sleep, rather than always trust to the rocking of the cradle, and the hush-hush droning of her own sweet voice.

There are, perhaps, even higher considerations than those of mere regard for health, that should induce mothers to have their children as much as possible in the open air. We know not, indeed, when the infant begins to notice the objects which it sees and hears, or how soon the impressions of good and evil, of love and hate, of sadness and joy are made upon its mind; but there cannot be a reasonable doubt, we think, that it is an observing, a remembering, perhaps even a reasoning, being long before it is generally imagined to be so. Hence the importance of surrounding children as frequently as possible with objects that, while they soothe, animate, while they gladden, elevate the mind; and where shall we find such objects so well as in the open fields and quiet woods?

But we must now draw our remarks on the Hygiene of early life to a close, and turn to the consideration of a few of the most important diseases to which children are exposed. We therefore take leave of the works of Dr. *Bull* and M. *Donné*—both of which are excellent guides in their way, and deserve to be generally known—and substitute for our text-books those of Mr. *Rees* and Dr. *Stewart*.

The first subject we shall notice, is the application of Auscultation—using this term in its widest acceptation—to the diagnosis of chest diseases in childhood.

Let us hear what our authors say.

According to Mr. *Rees*' experience—and our own is entirely in accordance with it—*Percussion* is of subordinate and only occasional use.

"It will not always yield that information in infants that it does in adults. A considerable portion of the lung may be rendered solid from inflammation in the former, and the chest not sound decidedly dull; and in cases where, after death, the lungs have been crowded with tubercles, I have failed to perceive any dulness on percussion. Nevertheless where hepatization is limited, as it mostly is, to the lower or upper lobes of one lung, percussion, if carefully performed, will detect it, even in infants, and with the auscultatory signs decide the diagnosis. In older children percussion is at times very valuable, for in them, as in adults, latent pneumonia proceeding to hepatization of a large portion of one lung is not uncommon; and in many cases, where the general symptoms have been vague and undefined, the breathing little affected, and the cough trifling, percussion of the chest has led to the detection of hepatization of the lung, and directed to the treatment likely to prove successful." *Rees*, 90.

As an instance of the uncertainty of any one means of diagnosis, taken *by itself*, he alludes to an interesting case of pleuritic effusion in a child, three years of age, where the chest on percussion gave out a tympanitic resonance, while no respiratory murmur was perceptible: death ensued in the course of a day or two. On dissection, the lung was found to be quite solidified; but the cavity of the pleura—which was much thickened from false membrane—was perfectly empty, shreds of lymph passing across. "In this case," says our author, "the effusion must have been very considerable, and have been rapidly absorbed, before the thorax had contracted from the dilated size to which the fluid had expanded it."

With respect even to *Auscultation*, the young physician must not expect to find that its indications are nearly so constant or so trustworthy for the purpose of diagnosis, in the young child as in the adult. It is obvious that Dr. *Stewart* does not speak from experience when he says that the respiratory sounds in general do not vary much in early life, and therefore do not require any particular description. Mr. *Rees* is, on the whole, much more judicious, as he expressly cautions his readers against the common error of attaching an undue importance to the auscultatory signs alone, especially in children's diseases, and without a sufficient attention to what have been called the 'rational symptoms.' Like every experienced practitioner, he does not allow one means of exploration ever to supersede the use of any other of established value.

In pneumonia in the child, the crepitant Rale is of rather uncertain occurrence. The sound is sometimes not to be heard at all; and when present, it does not always indicate the existence of pulmonic inflammation. Perhaps a more valuable sign, in the diagnosis of the early stage of the disease, is that first pointed out by Dr. *Stokes*: viz. an undue loudness of the respiratory murmur. Mr. *Rees* says; "I believe a knowledge of this fact will enable us to detect pneumonia in the child before other physical symptoms develop themselves." He proceeds to remark:—

"Where pneumonia has produced hepatization of any portion of the lung it will be readily recognized, though no dulness be present, by the existence of bronchial respiration and bronchophony, which are remarkably distinct in the young subject, the latter is at times so tinny and shrill as almost to simulate egophony. Dr. Meriadec Laennec, indeed, speaks with some doubt as to the facility of distinguishing bronchial respiration from peurile vesicular respiration, but the writer with great diffidence states, as the result of extensive experience, that the difference is most palpable, and that the former is to be recognized in many cases where a very small portion of the organ beneath is affected. I believe the cry of an infant, with a large portion of lung hepatized, has sometimes a peculiar clang, which will lead one to suspect the presence of such change of structure." *Rees*, 93.

On the whole, the utility of Percussion and Auscultation is unquestionably not nearly so great in the diseases of children as in those of adults; while, on the other hand, that of careful inspection of the thorax is decidedly greater. Much most useful information, in many cases of thoracic disease, may be obtained by attentively examining the shape and movements of the ribs—which in early life are, it is well known, more yielding and flexible than they afterwards become. When a considerable portion of the lung becomes solidified, the corresponding part of the chest will very generally be found more or less drawn in; and in this way not a few cases of what is called 'pigeon-chest' are induced.

"Again," says our author, "if the primary air-passage itself be much obstructed, as in croup, from false membrane, or from abscesses occurring under the sterno-mastoid and pressing the trachea, the full expansion of the lung will be prevented, and this altered movement of the ribs apparent. While these movements of the chest indicate the above affections, immobility of the chest is one remarkable symptom in the earliest stage of pleurisy in the infant and child, the respiration being wholly abdominal.

"But not only the movements of the chest are affected, but the shape is modified by disease of the lung; where, after pneumonia, the lung remains solid, it becomes after a time atrophied, and a contraction of the walls of the chest takes place, Dr. Stokes states, similar to that in pleurisy. I believe the contraction in the young child and infant is somewhat different; for where the lung in these has become atrophied, the contraction of the chest is effected by a falling in at the union of the ribs with their cartilages, giving the thorax at that part a channelled appearance." *Rees*, 96.

*Pleuritis* in young children is generally a sequela of scarlatina; and, as there is usually in such cases a marked tendency to other internal lesions —more especially to pericarditis and encephalic effusion—at the same time, the disease is unquestionably one of the most formidable that occurs in practice. The description of the symptoms given by Mr. *Rees* is very good:—

"The symptoms have been great dyspnœa, with short dry cough, and complaint of pain, as in the adult, with great fever. In the infant, such fever, with very rapid respiration, and constant teasing cough, have been the chief symptoms, with one other, which is the most characteristic, viz., extreme immobility of the chest in breathing, the respiration being wholly abdominal. When effusion results, the symptoms will be the same as in the adult, dulness, obliteration of intercostal depressions, enlargement of the affected side.

"But pleurisy may proceed in a very insidious manner in the child, and considerable effusion gradually take place, without any complaint of the chest being made by the patient. Twice I have met cases of this kind, where headache, with inability to move about, were all complained of; one of these was put down as hydrocephalus by the medical attendant. On examination, considerable effusion into the right pleura was discovered, for the discharge of which I performed paracentesis thoracis, and the child did well, contraction of the side resulting." *Rees*, 136.

Whenever, during the convalescence from scarlatina, the breathing becomes oppressed—and more especially if pyrexia be present, and the urine be scanty and deep-coloured at the same time—the physician should make a most careful examination of the thorax; for in nine cases out of ten it will be found that inflammation has commenced either in the pleura, lungs, or pericardium, or perhaps in all these parts simultaneously. The extreme rapidity with which the mischief, if not discovered and arrested, goes on to a fatal termination, cannot be too much impressed on the minds of all medical men. The delay of twenty-four hours will often suffice to convert a manageable into a desperate case; and this error is exceedingly apt to be committed, if there be not much feverishness present. The following graphic description of the onset and course of pericarditis after scarlatina evidently shews that it is drawn from personal observation:—

* * * "Before any active symptoms have developed themselves, if the stethoscope be applied over the heart, an alteration will be perceived in the sound, very distinct and characteristic, though not easily described; perhaps a ringing sound defines its character most accurately. After this is once heard, it will be easily recognized in another case, and may be put down as a sure symptom of incipient pericarditis. If this be neglected, in a few hours active symptoms come on; pyrexia, hurried breathing, palpitation, great irritability of temper, and restlessness, perhaps chorea; the pulse is full, thrilling, and irregular; on listening to the heart the ringing sound is found superseded by bruit de soufflet. This state continues for a day or two, and the symptoms again alter; the countenance becomes livid, the dyspnœa is excessive, accompanied with short convulsive cough, sometimes with hiccough; the pulse is weak, fluttering, and irregular; on listening to the chest, the heart's action is scarcely perceptible, the sound lost, and the impulse slight. On percussion, dulness over the left side will be perceived to a larger extent than natural; all showing effusion has taken place; and in a few hours the patient expires, often suddenly and without a struggle."* 217.

The main thing to do in such cases is to form a right diagnosis; the treatment is abundantly obvious, and will very generally be successful, if resorted to sufficiently early. Detraction of blood, locally or generally, followed by the application of blisters, and the free use of hydragogue purgatives, are our best remedies. The administration of some mercurial at the same time, so as to affect the system, is also of great utility in most cases. One of the least fallible symptoms to guide our prognosis is the condition of the urine. As long as this remains scanty and deep-coloured, we may rest assured that there is some internal mischief either impending or already established. Indeed this, the state of the urinary secretion, is the most important circumstance to be attended to in the management of scarlet fever; and whoever neglects his duty in this respect is exceedingly blameable. If the head be the part affected, when this condition of the urine is present, the danger is of the most imminent kind. We have known such cases prove fatal within 36 hours from the first invasion of the cephalic symptoms. This is the true *wasserschlag* of the German writers; and truly it may be called a *stroke,* from the frightful suddenness (apparent) of the attack, and the rapidity with which it hurries on to a fatal termination.

With respect to the treatment of pneumonic and bronchitic attacks in children, the only remark that we shall make is for the purpose of recommending most strongly the use of ipecacuan in frequently-repeated doses. A grain, with one or two grains of the hydrargyrum cum creta and as much carbonate of soda, may be given every four or six hours, according to the urgency of the symptoms; and in the intervals small doses of Epsom salts, nitre, and tincture of digitalis will be found to be of singular efficacy. Leeches must be applied to the chest—and immediately over the sternum is the right place on all occasions—as a matter of course; and, as the violence of the inflammation subsides, a blister between the shoulders should not be neglected.

### *Pathology of Laryngismus Stridulus.*

The personal experience of most medical men will fully warrant, we feel assured, the conclusion to which M. *Rees* has come on this subject; viz:

---

* The peculiar sound, to which Mr. *Rees* alludes in this passage, and which he very properly designates by the name of *ringing,* is the same, if we mistake not, which for many years past we have been in the habit of watching in cardiac diseases. The best idea that we can give of it is to say that it resembles the ringing noise or metallic resonance heard on striking an anvil with a light hammer. In our own note book, we have always described it as the 'metallic ringing.' It is a pretty constant symptom of the early stage of hypertrophy of the heart, especially if there be any slight narrowing of the aortic orifice at the same time.

that the cause of this peculiar affection is by no means uniformly the same in all cases; but that the 'fons et origo mali' may be seated either in the encephalon, in the thymus gland, or in the bronchial and cervical glands, as alleged by different authors.

"I have had many opportunities," says he, "of examining infants who have died from this affection. Among infants, otherwise apparently healthy, who have been suddenly cut off in a paroxysm of the child crowing, the most prevalent abnormal appearance has been hypertrophy of the thymus gland, more or less considerable, and in these infants I have failed to perceive any enlarged glands in the cervical region, though I have searched for them; the thymus gland has been of considerable size, weighing five, six, or even seven drachms.* In other cases, where there has been during life cerebral symptoms associated with the spasmodic dyspnœa, there has been no such enlargement of the thymus, but the morbid appearances were confined to the brain and its meninges; opacity of the arachnoid, thickening of the same at the base and over the pons varolii, with considerable injection of the hemispheral veins, and numerous bloody points in the brain, being the chief morbid appearances. In some of these I have met with enlargement of the cervical and bronchial glands, and such I have found in infants who suffered with laryngismus stridulus during life, where neither hypertrophy of the thymus, nor any marked unhealthy appearances in the brain have been detectible. From the cases quoted by Dr. H. Ley, it is certain an enlargement of these glands is a very frequent post-mortem appearance. From what I have seen I cannot but imagine that no one post-mortem appearance is so constant as to justify us in considering the part presenting it the seat of the affection in all cases; and it may be inquired, if the spasm be induced, as Dr. H. Ley supposes, by irritation of the recurrent nerves, which seems so probable, whether the exciting cause may not be different in different cases. An hypertrophied thymus gland, or enlarged cervical or bronchial glands, may alike directly or indirectly by pressure embarrass the function of that nerve, nor does it seem difficult to imagine the spasm in other instances may be excited by cerebral disease situated at the base of the brain, and implicating the origin of the par vagum." *Rees*, 116.

*Intestinal Disorders; Remittent Fever, &c.*

So many of the diseases of children are connected with an unhealthy state of the intestinal contents, that, unless the medical attendant has his attention constantly directed to this subject—and, by the bye, he can rarely form an accurate opinion except by personal inspection of the evacuations—he will be often baffled in his best attempts to afford relief. We have almost daily occasion in our own practice to witness the effects of mismanagement in this respect, and not unfrequently to regret the negligence, or it may be the ignorance, of our professional brethren on the subject.

As a general remark, we may say that two thirds, or even three fourths, of the diseases of children arise from the presence of undigested food and vitiated secretions in the bowels; and that, unless the canal be thoroughly cleansed out, no permanent cure can be effected. Almost every case of what is called 'infantile remittent fever' arises from the cause now mentioned. In obstinate cases, the necessary purgation must be continued for two,

---

* "The largest weighed ℥viiss. See letter by the author in the Lancet for May 22, 1841."

three, or even four weeks. Like every other mode of treatment indeed, this may be carried too far; and a state of intestinal irritation, as troublesome as the primary evil, may be induced in consequence.

There is much good sense and practical usefulness in the following observations of Mr. *Rees*. After urging the necessity of free purgation and a spare regulated diet, as long as the intestinal excretions are unhealthy and offensive, and there is considerable feverishness of the system, he points out an error, not unfrequently committed, of carrying the use of such means too far, merely because the tongue has not quite regained its healthy appearance, and an occasional feverishness of the system still remains behind.

"I believe," says he, "the most important therapeutic agent to be recommended, when such a state is present, is change of air, and this may prove of marked benefit in a very short time; the mucous membrane augmenting its secretions, and being thus enabled to digest the food, which, when administered before, acted as an irritating matter. Another most important agent is the warm bath; this may be used every evening, the patient remaining in ten minutes, and transferred to a warm blanket; this often exerts a powerful influence in restoring the mucous surfaces to healthy secretion. Of internal remedies, perhaps, the most useful will be the hyd. c. cret., given in small doses every night for some little time; for an infant, a grain, or two grains, may be sufficient; this medicine will, at the same time that it acts beneficially on the system generally, have the effect of checking diarrhœa, which is often present. All active purgatives should be given as sparingly as possible; and the bowels kept regular by a dose of castor oil, or rhubarb and magnesia, every, or every other, morning.

"The great error, in the treatment of these children, is the endeavour to overcome the debility by stimulants, or tonics; the infant has wine and water administered, or the child is recommended full diet, and a little stout or wine; whereas, to give other than a very mild diet is to increase the debility by augmenting the irritation, which fact, though it appear plain to the reader, he will find it difficult so to explain and impress on the mind of the parent, that when his back is turned, his rules of diet shall not be forgotten. It will be, therefore, necessary to lay down very explicit rules. In the infant, irritation may be prolonged by the common practice of stopping every expression of suffering by thrusting the nipple into its mouth." *Rees*, 28.

We have only to add to these excellent simple rules that, in many such cases, not a little benefit will be obtained by keeping the head of the child uniformly cool—wetting it frequently with cold water during the day, and exposing it uncovered at night—and by the internal administration of small doses of *nitre* in conjunction with a few drops of tincture of digitalis. In larger doses, the nitre is a capital remedy in the early stage of febrile diarrhœa; it quiets the intestinal irritation, without directly checking the evacuations. If these continue to be too profuse, a few spoonfuls of the common chalk mixture will generally suffice to arrest them without danger. The less frequently that we make use of opium and of direct astringents in the diarrhœa of young children, the better: the Homœopathic doctrine—"similia similibus curantur"—is, as our author remarks, of safer application on the whole, at least in a great many cases. On several occasions, we have seen serious cephalic symptoms set in immediately upon the cessation of a relaxed state of the bowels, more especially if the irritation of teething was present at the same time. It is for this reason that we are so partial to the use of nitre in many cases of febrile diarrhœa in children.

We proceed now to notice a disease of rare occurrence in this country, but which is exceedingly prevalent and very destructive in most parts of America: viz:

*Cholera Infantum.*

"It has been said," Dr. Stewart remarks, "by some of our writers, to be peculiar to the United States. This, however, appears to be an error, for Cleghorn, in his account of the diseases of Minorca, describes a disease, bearing symptoms in every respect corresponding to those of this disease.

"Wherever, therefore, there is great atmospheric heat, combined with malaria, and especially with that engendered by large collections of inhabitants, as in cities, there the disease is found. It is on this account extensively prevalent in our country; but is not dependent on heat alone, as it is observed that in some of our most southern sections of country it is not so rife as in New-York or Philadelphia. The open country is remarkably exempted from this scourge.

"It prevails mostly in cities, and is in some places a remarkably fatal disease. In Philadelphia it is much more fatal than in New-York or Boston; and in the latter city it has been doubted by some whether the disease exists in its genuine form."—*Stewart*, 213.

The irritation of teething and the use of improper food are the *predisposing*, but cannot be said to be the directly *exciting*, causes of this disease, as it is comparatively of rare occurrence in the rural districts. The Malaria of large towns must therefore have something to do with its development. The description of the purging is thus given by Dr. *Stewart.*

"For the most part, the diarrhœa will commence with the discharge of ordinary fecal matter, which very soon, however, changes to a thin, serous looking fluid, variously coloured. When the disease is fully formed, the ordinary feces are retained, and the evacuations are mostly of this watery fluid, coloured brown white, sometimes yellow and green. They are at times very offensive, and again without any other odour than what arises from acidity. In the greatest number of instances they are offensive, but not of the peculiar character which distinguishes fecal matter; it appears to be of the nature which characterizes the ordinary decomposition of animal matters. According to the location of the disease in different parts of the intestinal tube, the evacuations present a difference in their consistency and appearance; thus, when the lower portion of the intestines is affected, the disease bears a resemblance to dysentery, and the evacuations are slimy, gelatinous, and bloody; while tenesmus and pain, on evacuting the contents of the bowels, are very severe. In the most severe cases, the evacuations show the extent of the disease throughout the entire intestinal tract; and it is in such cases that they present a great difference in their appearance, including all the above varieties, and at times mixed with a frothy matter resembling yeast, and with imperfectly-digested food, or that which has passed almost unaltered."—*Stewart*, 216.

The vomiting is often a most unconquerable symptom. The average duration of the disease appears to be from two to three weeks. The essential necroscopic characters are, according to our author, engorgement and often very great enlargement of the liver, with the usual consequences of inflammation along the whole intestinal canal, more especially in the mucous follicles of the small bowels.

It is unnecessary to enter into particulars about the treatment, as the same therapeutic principles, which regulate our practice in other forms of cholera, are equally applicable in this. We should say however that Dr. *Stewart* is far too much afraid of the consequences of Emetics—which, if administered at first, are, in our opinion, most useful not only in dislodging a great quantity of vitiated bile from the stomach and bowels, but even in checking the very vomiting, which is so distressing a symptom of the disease. Mercurials are, it is admitted by almost all the American authors, more or less necessary in every case. One of the least objectionable astringents is the acetate of Lead, alone, or in combination with Dover's powder. Dr. *Lindsley*, of Washington, has given as much as four grains of the former and one of the latter every hour or two to a child eighteen months old. During the period of convalescence, the use of bitter infusions, of steel and other tonics, is usually necessary for some time. It has been very generally observed, that children crave much for salted meat and fish, while recovering from this disease; and the most experienced physicians, including Drs. *Rush, Parrish,* and *Hosack,* have remarked that this instinct may be indulged not only with perfect safety, but with positive advantage to the health.

With this observation we close the present article. We have made it as practical as possible, and may therefore hope that it will be perused with some interest by most of our readers. The work of Dr. *Stewart* is not likely to acquire much circulation in this country, as it is rather a lengthened compendium of what has been written by others, than a summary of the results of his own observation and experience. That of Mr. *Rees*, although less complete as a treatise on the subject, is far more original; it is evidently written by a man who has seen much practice, and learned to observe and judge for himself.

---

Brighton and its Three Climates, &c. &c. &c. By *A. L. Wigan*, M.D. Octavo, pp. 71. Highley, 1843.

